# Modelling of Mechanical Response of Weldlines in Injection-Moulded Short Fibre-Reinforced Polymer Components

**DOI:** 10.3390/polym17192712

**Published:** 2025-10-09

**Authors:** Matija Nabergoj, Janez Urevc, Miroslav Halilovič

**Affiliations:** 1 CAFUTA d.o.o., Kalce 5j, 1370 Logatec, Slovenia; matija.nabergoj@cafuta.eu; 2 Faculty of Mechanical Engineering, University of Ljubljana, Aškerčeva 6, 1000 Ljubljana, Slovenia; janez.urevc@fs.uni-lj.si

**Keywords:** short-fibre-reinforced polymers, weldline modelling, damage, fibre orientation, injection moulding, process simulation, constitutive modelling, digital image correlation

## Abstract

Short fibre-reinforced polymers (SFRPs) are increasingly used in structural applications where mechanical integrity under complex loading is critical. However, conventional modelling approaches often fail to accurately predict mechanical behaviour in weldline regions formed during injection moulding, where microstructural anomalies and pre-existing damage significantly degrade performance. This study addresses these limitations by extending a hybrid micro–macromechanical constitutive framework to incorporate localised initial damage at weldlines. Calibration and validation of the model were conducted using directional tensile tests on dumbbell-shaped polyamide 66 specimens reinforced with 25 wt% glass fibres, featuring controlled weldline geometry. Digital image correlation (DIC) was employed to capture strain fields, while injection moulding simulations provided fibre orientation distributions and weldline positioning. Results demonstrate that incorporating initial damage and its independent evolution for the cold weld region significantly improves prediction accuracy in weldline zones without compromising model efficiency. The proposed approach can be integrated seamlessly with existing finite element framework and offers a robust solution for simulating SFRP components with weldlines, enhancing reliability in safety-critical applications.

## 1. Introduction

Short fibre-reinforced polymers (SFRPs) are widely used in industries such as automotive, electronics, biotechnology, and defence [1]. Their high strength-to-weight ratio, good dimensional stability, and robust thermomechanical properties make them particularly suitable for injection moulding of geometrically complex components at low cost and high throughput [2,3,4].

Originally used in less demanding parts, SFRPs are now increasingly employed in load-bearing components exposed to cyclic mechanical loading and harsh environments [4]. Finite element method simulations, enhanced with constitutive models, are routinely used to predict the mechanical response of such components. In this context, a critical parameter is the fibre orientation distribution (FOD), which governs the anisotropic stiffness and strength of the material [5].

Although this modelling approach performs well in homogeneous regions, it becomes unreliable in areas where the microstructure and quality of the material are locally altered, such as in cold weld regions [6]. These are formed when two melt fronts meet at angles less than 135° [7]—typically due to multiple injection points or geometric features of the mould [8]. In such regions, reduced molecular interdiffusion, voids, notches on the surface, and disrupted fibre alignment result in significantly degraded mechanical properties—up to 50% lower than in base material [6,8,9].

Cold welds are not only a common source of mechanical weakness but, in some applications, are intentionally used as controlled failure locations. In automotive and safety-critical applications, their placement can define energy-absorbing zones that protect critical components during overload events [10]. In such cases, reliable prediction of local mechanical behaviour is essential for both durability and functional safety. Yet, conventional modelling approaches typically consider only the effect of fibre orientation, ignoring other key physical phenomena that govern the structural integrity of weldlines.

Experimental observations have repeatedly shown that in cold weld regions, a reduction in stiffness is already present at the onset of loading. This suggests the presence of pre-existing damage or structural anomalies that are not captured by orientation-based stiffness models alone. These effects—poor molecular bonding, voids, surface notches, and molecular alignment perpendicular to flow—act as damage precursors, degrading the material’s ability to carry load.

To improve prediction accuracy, researchers have taken various approaches. Some models neglect fibre–matrix interaction and assume weld strength equals matrix strength reduced by fibre content [11], while commercial packages often apply empirical weakening factors that must be calibrated for each geometry and process window [12,13]. More advanced approaches include data-driven algorithms [7] and extensions based on reptation theory to model diffusion and entanglement across the weld [6,14].

While promising, these models have limitations. For example, Cruz’s model [6] showed good agreement with experiments in unreinforced thermoplastics, but large deviations persisted for fibre-reinforced materials, which were attributed to the inaccurate prediction of fibre orientation at the weld [15]. Baradi’s study [16] revealed that even with accurate fibre orientation input achieved by X-ray computed microtomography, the predicted stress–strain response failed to match the experimental one at the weld, indicating that altered damage behaviour should also be considered.

These findings demonstrate that accurately modelling the mechanical behaviour of cold welds requires accounting not only for local fibre orientation, but also for the other mentioned phenomena that contribute to the softening of the weld’s mechanical response. An elegant way to encapsulate all of them in a constitutive model, without significantly enlarging the number of model parameters, is to consider them as initial damage, as will be performed in this work.

Currently, micromechanical models based on the mean-field method [17,18] are most prevalent, offering the advantage of incorporating microscale physical mechanisms and enabling phase-specific stress–strain tracking. However, their high computational cost limits practical application. On the other hand, fully macromechanical models [19,20,21,22,23,24] adopt a phenomenological approach throughout, significantly reducing computational demands and enhancing industrial applicability. A reasonable balance between accuracy and efficiency is represented by combined micro–macromechanical models [25,26,27], which typically approximate the initial stiffness via mean-field methods and treat other phenomena phenomenologically.

Taking this into account, we adopted and extended a recently proposed hybrid micro–macromechanical model by Schulenberg et al. [27]. This model combines the following: (i) an analytical mean-field homogenisation (Mori–Tanaka, extended using Advani–Tucker orientation tensors) for predicting anisotropic elastic stiffness based on fibre orientation, and (ii) a phenomenological formulation for nonlinear plasticity and damage, applied along principal fibre axes. Originally developed for long-fibre thermoplastics, this model is particularly suitable for SFRPs as well, due to its modularity and efficiency. It has already been successfully applied to a simulate punch test, demonstrating its capability to capture complex loading scenarios. Its structure allows straightforward adaptation of damage behaviour in specific sub-regions—such as the cold weld—without altering the general framework or the base material model, as will be demonstrated in this work. This represents a novel application, since the model has not previously been employed in weldline regions.

In this study, we make use of the modular structure of the Schulenberg et al. [27] model to model the cold weld region as an initially damaged subdomain. This approach enables us to encapsulate the cumulative effects of various phenomena—such as poor molecular bonding, void formation, surface notches, and molecular alignment perpendicular to the flow direction—that compromise the structural integrity of the weld beyond the influence of FOD. For model calibration and validation, we designed a targeted experimental procedure using dumbbell-shaped injection-moulded specimens from polyamide reinforced with 25 wt% glass fibres with a centrally located cold weld. This geometry ensures controlled processing conditions and a simplified flow field, which facilitates accurate simulation of fibre orientation. In addition, it allowed us to isolate four different specimens with specific fibre orientations for tensile mechanical testing—parallel and perpendicular to the fibres in the base (weldline-free) material and parallel and perpendicular to the weldline. With the results of directional tensile tests (monotonic and cyclic loading), we were able to calibrate specific model parameters to specific measured material response. Plasticity parameters were calibrated to monotonic tests of the base material, damage parameters were calibrated to the results of the cyclic tests of the weldline-free material, and initial damage on the weld area was calibrated to the cyclic tests perpendicular to the weld. The measurements of the mechanical response parallel to weld direction were then used for validation of the calibrated parameters. Results confirm that extending models with initial damage can be a robust and efficient pathway. This extension enables the inclusion of structural phenomena present in the weld region, thereby improving weldline prediction in structural SFRP components.

## 2. Materials and Methods

### 2.1. Theoretical Preliminaries—Description of the Implemented Numerical Model

Schulenberg et al. [27] have introduced a mathematical model combining an analytical micromechanical model for elastic properties with a phenomenological approach for plastic behaviour and damage. The Mori–Tanaka method [28] is used for efficient one-step homogenisation of the linear elastic stiffness tensor, extended to account for local FOD using the Advani–Tucker approach [5]. Nonlinear plastic behaviour is homogenised separately through a weighted average over the principal FOD directions, applying Hill’s yield criterion. Furthermore, damage evolution is also well accounted for phenomenologically. In the following, we summarise details of the model with an emphasis on presenting our addition of initial damage at the weld area.

#### 2.1.1. Effective Stiffness Tensor

##### Mori–Tanaka Homogenisation

The Mori–Tanaka method is based on Eshelby’s solution for an ellipsoidal inclusion embedded in a linear elastic matrix [29]. Using the concept of an equivalent inclusion, the inhomogeneity is treated as a separate material embedded in a matrix. The macroscopic strain $\bar{\varepsilon }$ is given as follows:

(1) $$\bar{\varepsilon }=c^{i}\varepsilon^{i}+\left(1-c^{i}\right){\bar{\varepsilon }}^{m},$$

where $c^{i}$ is the volume fraction of the inclusion, $\varepsilon^{i}$ is the strain in the inclusion, and ${\bar{\varepsilon }}^{m}$ is the average matrix strain. To relate the inclusion strain to the overall strain, a strain localisation tensor L is introduced:

(2) $$\varepsilon^{i}=L:\bar{\varepsilon },\;L={\left[I+E:C^{m^{-1}}:\left(C^{i}-C^{m}\right)\right]}^{-1},$$

where $C^{i}$ and $C^{m}$ are stiffness tensors of the inclusion and matrix, E is Eshelby tensor, dependent on the inclusion’s aspect ratio and matrix Poisson’s ratio $v_{m}$. In the Mori–Tanaka scheme, localisation relates to the following matrix strain:

(3) $$\varepsilon^{i}=L:{\bar{\varepsilon }}^{m},$$

The effective stiffness tensor $C^{*}$ is then as follows:

(4) $$C^{*}=\left[c^{i}C^{i}:L+\left(1-c^{i}\right)C^{m}\right]:{\left[c^{i}L+\left(1-c^{i}\right)I\right]}^{-1}.$$

Assuming a prolate ellipsoidal inclusion (fibre-like), the result is a transversely isotropic effective stiffness tensor.

##### Tensorial Description of Fibre Orientation Distribution

For practical applications, especially with local fibre orientation data from mould filling simulations, a continuous function is often not ideal. A tensorial representation with discrete values, as proposed by Advani and Tucker [5], is more effective. In this method, a unit vector $p\left(\theta ,\phi \right)$ is used to represent fibre direction in spherical coordinates. All orientations lie on the half of the unit sphere, where $\left(\theta ,\phi \right)\epsilon \left[0,\pi \right]$.

The second- and fourth-order fibre orientation tensors are defined by integrating the dyadic products of the unit vectors p weighted by the orientation distribution function $\psi \left(\theta ,\phi \right)$:

(5) $${\left[A\right]}_{ij}=A_{ij}=\oint \;p_{i}p_{j}\psi \left(p\right)dp$$

(6) $${\left[A\right]}_{ijkl}=A_{ijkl}=\oint \;p_{i}p_{j}p_{k}p_{l}\psi \left(p\right)dp$$

##### Orientation-Averaged Effective Stiffness

The anisotropic behaviour of a material can be described using the FOD [5]. The transversely isotropic stiffness tensor $C^{*}$ can be orientation-averaged, denoted as ${\bar{C}}^{*}$, using tensors A and A as follows:

(7) $${\bar{C}}_{ijkl}^{*}=b_{1}\;A_{ijkl}+b_{2}\left(A_{ij}\delta_{kl}+A_{kl}\delta_{ij}\right)+b_{3}\left(A_{ik}\delta_{jl}+A_{il}\delta_{jk}+A_{jl}\delta_{ik}+A_{jk}\delta_{il}\right)+b_{4}\delta_{ij}\delta_{kl}+b_{5}\left(\delta_{ik}\delta_{jl}+\delta_{il}\delta_{jk}\right).$$

Here, the five scalars $b_{1}$ to $b_{5}$ correspond to the independent components of the transversely isotropic stiffness tensor ${\bar{C}}_{ijkl}^{*}$, with the 1-direction as the principal axis of anisotropy. Using Voigt’s notation (11 → 1, 22 → 2, 33 → 3, 12 → 4, 23 → 5, 13 → 6), the scalar coefficients are defined as follows [5]:

(8) $$\begin{array}{c} b_{1}=C_{11}^{*}+C_{22}^{*}-{2C}_{12}^{*}-{4C}_{44}^{*}\\ b_{2}=C_{12}^{*}+C_{23}^{*}\\ b_{3}=C_{44}^{*}+\frac{1}{2}\left(C_{23}^{*}-C_{22}^{*}\right)\\ b_{4}=C_{23}^{*}\\ b_{5}=\frac{1}{2}\left(C_{22}^{*}+C_{23}^{*}\right) \end{array}$$

##### Closure Approximation

In many cases, especially in mould filling simulations, fibre orientation data are only available as a second-order orientation tensor. However, to use the orientation-averaged stiffness model, a fourth-order tensor A is needed. Here, the original Schulenberg et al. [27] model uses hybrid closure approximation [5] which is computationally efficient, but may give inaccurate results in intermediate or biaxial states of orientation. Instead we decided to use IBOF closure approximation [30] which can be highly accurate in a wide range of anisotropies. General expression of a full symmetric fourth-order tensor $A_{ijkl}$ in terms of $A_{ij}$ and $\delta_{ij}$ is as follows:

(9) $$\begin{array}{c} A_{ijkl}=\beta_{1}S\left(\delta_{ij}\delta_{kl}\right)+\beta_{2}S\left(\delta_{ij}A_{kl}\right)+\beta_{3}S\left(A_{ij}A_{kl}\right)+\beta_{4}S\left(\delta_{ij}A_{km}A_{ml}\right)+\beta_{5}S\left(A_{ij}A_{km}A_{ml}\right)\\ \;\;\;\;\;\;+\beta_{6}S\left(A_{im}A_{mj}A_{kn}A_{nl}\right) \end{array}$$

where the operator *S* indicates the symmetric part of its argument. For clarity of the text, the remaining equations and explanations on IBOF implementation can be found in the referenced article.

#### 2.1.2. Approximation of Plastic Material Behaviour

Plasticity in the matrix material is often modelled using incremental homogenisation and tangential stiffness calculations, as shown in studies by Doghri et al. [31,32,33,34] and Nguyen et al. [35,36,37]. While accurate, these methods lead to high computational costs in explicit finite element simulations due to small time step requirements. To address this, the Schulenberg et al. model adopts a more phenomenological approach, decoupling plasticity from the linear elastic homogenisation scheme.

**Figure 1:**
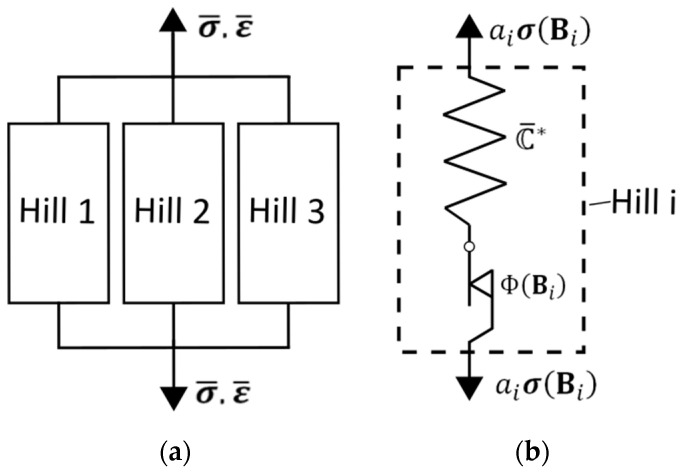
Rheological network of the material model: (**a**) The rheological network connects three Hill’s elastic–plastic models in parallel; (**b**) Each parallel branch contains a Hill’s yield criterion and is loaded with a weighted stress $a_{i}\sigma \left(B_{i}\right)$.

Hill’s transversely isotropic yield criterion is applied three times in parallel (Figure 1), each corresponding to a principal fibre orientation defined by the second-order orientation tensor A. The overall stress response is obtained through a weighted average of the three branches, using the eigenvalues ($a_{1},a_{2},a_{3}$) of A as weights. This orientation-averaged elasto–plastic formulation significantly enhances computational efficiency.

##### Hill’s Yield Criterion

For unidirectional fibre orientations, the material exhibits transversely isotropic behaviour. In this case, Hill’s yield criterion [38] is well-suited. To incorporate fibre direction into the plasticity model, an orientation tensor B is defined using the dyadic product as follows:

(10) $$B=e^{*}⊗e^{*}$$

where the unit vector $e^{*}$ denotes the fibre direction. Based on Aravas [39], Hill’s yield criterion can be expressed using tensor B as follows:

(11) $$\Phi \left(\sigma ,B\right)=\left(G+2F\right)tr\left(\sigma^{\prime 2}\right)+2\left(M-G-2F\right)tr\left(\sigma^{\prime 2}\cdot B\right)+\left(5G+F-2M\right){tr}^{2}\left(\sigma^{\prime }\cdot B\right)-\sigma_{y}^{2}\left(\varepsilon_{p}\right)$$

Here, $\sigma_{y}$ is the yield stress, $\sigma^{\prime }$ is the deviatoric part of stress tensor σ, and F,G,M are parameters that characterise anisotropy. For F=G=0.5 and M=1.5, the model simplifies to the isotropic von Mises criterion. The hardening law depends on the effective plastic strain $\varepsilon_{p}$ and is given as follows:

(12) $$\sigma_{y}\left(\varepsilon_{p}\right)=\sigma_{0}+h\varepsilon_{p}^{q}$$

where $\sigma_{0}$, *h* and *q* are material parameters. The plastic strain rate tensor is defined using the normality flow rule:

(13) $${\dot{\varepsilon }}_{p}=\dot{\lambda }\frac{\partial \Phi \left(\sigma ,B\right)}{\partial \sigma }$$

where $\dot{\lambda }$ is a plastic multplier.

##### Orientation-Averaged Plasticity Model

To incorporate arbitrary FODs into the plasticity model, a simplified averaging approach is applied. The principal fibre directions correspond to the eigenvectors $e_{i}^{*}$ of the fibre orientation tensor  A:

(14) $$A=\sum_{i=1}^{3}\sum_{j=1}^{3}A_{ij}e_{i}⊗e_{j}=\sum_{i=1}^{3}a_{i}e_{i}^{*}⊗e_{i}^{*}$$

where $e_{i}$ are unit vectors in the global coordinate system and $a_{i}$ (with $\sum_{i=1}^{3}a_{i}=1;a_{i}\epsilon \left[0,1\right]$) are the eigenvalues representing the FOD along direction $e_{i}^{*}$. Following the rheological model (Figure 1), three yield criteria $\Phi \left(B_{i}\right)$ are defined using orientation tensors $B_{i}=e_{i}^{*}⨂e_{i}^{*}$. Solving $\Phi \left(B_{i}\right)\leq 0$ yields the corresponding stress tensors $\sigma \left(B_{i}\right)$. The effective stress tensor $\bar{\sigma }$ is calculated as a weighted average using $a_{i}$:

(15) $$\bar{\sigma }=a_{1}\sigma \left(B_{1}\right)+a_{2}\sigma \left(B_{2}\right)+a_{3}\sigma \left(B_{3}\right)$$

Here, $\sigma \left(B_{i}\right)$ denotes stress tensors dependent on $B_{i}$. No explicit orientation-averaged yield surface is used; instead, the model averages the response of three elasto–plastic models aligned with principal fibre directions. The same Hill parameters (***F***, ***G***, ***M***) are applied across all three branches.

#### 2.1.3. Incorporation of Damage and Failure

During unloading, we can distinguish and evaluate irreversible effects based on whether the material response is attributed to damage or plasticity. For numerical implementation, Voigt’s notation is employed, representing the symmetric Cauchy stress tensor σ as a six-dimensional vector ${\left[\sigma \right]}_{\alpha }\;\left(\alpha =1,\ldots ,6\right)$. Due to the complexity of interactions between loading and damage, six independent damage variables $d_{\alpha }$ are introduced, allowing component-wise damage tracking. The resulting damage effect tensor M is defined as follows [40]:

(16) $$M=\left[\begin{array}{cccccc} 1-d_{1} & 0 & 0 & 0 & 0 & 0\\ 0 & 1-d_{2} & 0 & 0 & 0 & 0\\ 0 & 0 & 1-d_{3} & 0 & 0 & 0\\ 0 & 0 & 0 & 1-d_{4} & 0 & 0\\ 0 & 0 & 0 & 0 & 1-d_{5} & 0\\ 0 & 0 & 0 & 0 & 0 & 1-d_{6} \end{array}\right]$$

The damaged stress components are computed by reducing the undamaged stress $\sigma_{\alpha }$ using the following formula:

(17) $$\sigma_{\alpha }^{dam}=\left(1-d_{\alpha }\right)\sigma_{\alpha }$$

In tensor form, this can be written as:

(18) $$\sigma^{dam}=M\sigma$$

Initially, all $d_{\alpha }=0$ (undamaged). Failure occurs when any $d_{\alpha }$ reaches value 1. Damage evolution is based on the maximum historical strain $\varepsilon_{\alpha ,max}$, with damage variables given as follows:

(19) $$d_{\alpha }={\left(\frac{\varepsilon_{\alpha ,max}}{\varepsilon_{f}}\right)}^{g}$$

Here, $\varepsilon_{f}$ is the failure strain and *g* is the damage evolution exponent. For simplicity, $\varepsilon_{f}$ and *g* are assumed identical for all components, which is sufficient when derived from tensile tests.

#### 2.1.4. Modelling of Mechanical Response of the Weld Area

Having provided an overview of the existing numerical model [27], we now introduce our approach to modelling of mechanical response in the weld area. As presented in the introduction, there are multiple reasons that can cause softer response of the material at the weld, while the model in its current state can account only for the FOD. The most intuitive way to soften the mechanical response of the modelled material without changing any of the parameters is to use initial damage. Simultaneously, the reduction in material integrity must be confined to the region affected by the weld, whereas the remainder of the component must retain the damage level $d_{\alpha }$, as defined in Equation (19). To enforce this condition, a localised damage $d_{\alpha ,weld}$ is assigned at the centre of the weldline. In the adjacent zones extending a distance $x_{weld}$ in both directions, the damage distribution is interpolated linearly using first-order Lagrange polynomials $\psi_{1}$ and $\psi_{2}$, as defined in Equation (20).

(20) $$d_{\alpha ,new}(x)=\left\{\begin{array}{cc} d_{\alpha ,weld}\psi_{1}+d_{\alpha }\psi_{2}=d_{\alpha ,weld}\left(1-\frac{\left|x\right|}{x_{weld}}\right)+d_{\alpha }\left(\frac{\left|x\right|}{x_{weld}}\right), & \left|x\right|\leq x_{weld}\\ d_{\alpha }, & \left|x\right|>x_{weld} \end{array}\right.$$

**Figure 2:**
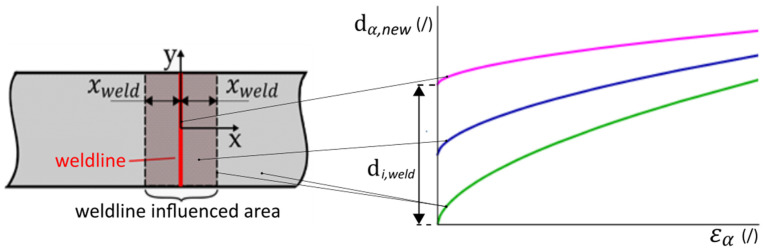
Damage at the weldline-influenced area; on the left side—positioning of the coordinate system and presentation of the weldline-influenced area, on the right side—schematic presentation of the damage evolution across the weldline-influenced area according to Equation (20).

In analogy to $d_{\alpha }$ defined in Equation (19), we now define $d_{\alpha ,weld}$ as follows:

(21) $$d_{\alpha ,weld}=d_{i,weld}+{\left(\frac{\varepsilon_{\alpha ,max}}{c_{weld}}\right)}^{g},$$

where $d_{i,weld}$ denotes the initial damage localised at the centre of the weld and $c_{weld}\;$ represents the damage evolution parameter specific to the weld-affected zone. In Figure 2, we present this schematically. By positioning the weld at the origin of the coordinate system, a spatially resolved damage model is established. In regions sufficiently distant from the weld, where the material is assumed to be initially undamaged, damage accumulation occurs solely as a function of deformation and is absent at the initial state (green line). Conversely, within the weld region, damage is present from the onset and evolves according to its own kinetics. A continuous transition across the defined boundary ensures a smooth interpolation between the two damage regimes. The magenta line presents values of the damage at the centre of the weldline, while values approximately in the middle of the weldline-influenced area are presented by a blue line. This formulation enables the simulation of inherent defects such as initial voids or suboptimal molecular entanglement characteristic of the weld zone, while preserving the integrity of the surrounding base material.

#### 2.1.5. Calibration Procedure

**Figure 3:**
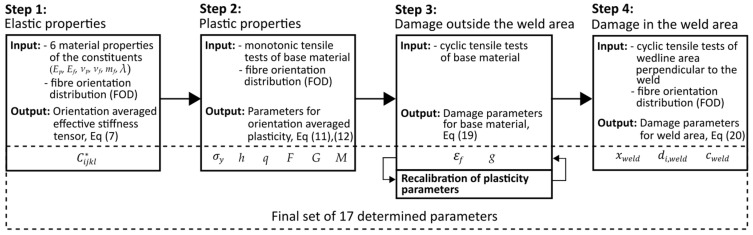
Model parameter calibration workflow; output presents the determined parameters of the model.

The numerical model under consideration comprises 17 parameters, rendering inverse identification a highly complex task in the absence of a carefully structured calibration procedure. Conveniently, these parameters can be systematically decoupled and calibrated in a stepwise manner. Based on the physical phenomena they represent, the parameters can be classified into four distinct categories: elasticity, plasticity, damage outside the weld area, and initial damage specific to the weld region. Figure 3 illustrates the calibration workflow, accompanied by the corresponding experimental input data required for each step of the procedure.

The calibration process begins with the computation of the orientation-averaged effective stiffness tensor ${\bar{C}}_{ijkl}^{*}$, utilising the Mori–Tanaka homogenisation scheme in conjunction with Advani–Tucker orientation averaging. This step requires fibre orientation distribution (FOD) data obtained from moulding simulations, as well as the material properties of the individual constituents—namely, the polymer and fibre. These include the elastic moduli (*E_p_*, *E_f_*), Poisson’s ratios (*ν_p_*, *ν_f_*), the fibre mass fraction (*m_f_*), and the fibre aspect ratio (*λ*). All constituent properties are provided by the material suppliers.

In the second step, starting estimates of six plasticity parameters are calibrated: three hardening parameters ($\sigma_{y}$, *h*, *q*) and three Hill’s yield criteria parameters (*F*, *G*, *M*). As damage effects are neglected at this stage, only monotonic tensile tests of the base material are required. These tests must be conducted both parallel and perpendicular to the fibre orientation to ensure accurate calibration of the Hill’s parameters.

The third step incorporates damage modelling of the base material, as described by Equation (19), which introduces two additional parameters: the failure strain ($\varepsilon_{f}$) and the damage evolution exponent (*g*). Since damage is characterised by the degradation of elastic properties, cyclic loading–unloading tests are necessary. Due to inclusion of the damage, which affects the plasticity response, recalibration of the previously determined plasticity parameters needs to be performed (mostly the three hardening parameters $\sigma_{y}$, *h*, *q*).

In the final calibration stage, Equation (19) is replaced by Equation (20), which extends the damage model to include the weld region. This introduces three new parameters: the weldline-influenced area ($x_{weld}$), initial weld damage ($d_{i,weld}$), and weld damage evolution coefficient ($c_{weld})$. The parameter $x_{weld}$ is determined from FOD data, while $d_{i,weld}$ and $c_{weld}$ are calibrated by fitting the model to cyclic tensile tests performed on weldline specimens in direction perpendicular to the weldline.

Upon completion of this final step, the calibration procedure yields a comprehensive set of 17 parameters.

### 2.2. Experimental Investigation

**Figure 4:**
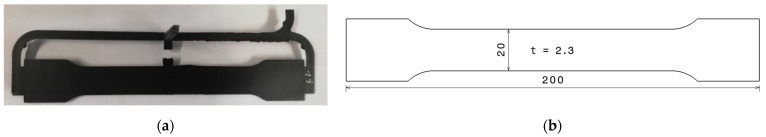
Sample: (**a**) Injection-moulded sample; (**b**) dimensions [mm].

For experimental investigation, dumbbell specimens with 20 mm × 2.3 mm cross-sections, presented in Figure 4, were fabricated using polyamide 66 reinforced with 25 wt% glass fibres (Solway Technyl A20 V25 Black). Specimens were injection-moulded from both sides, creating a cold weld in the centre of the specimen. For injection moulding, Kraus Mafei 0050/00180/CX/06 machine was used with the parameters presented in Table 1.

**Figure 5:**
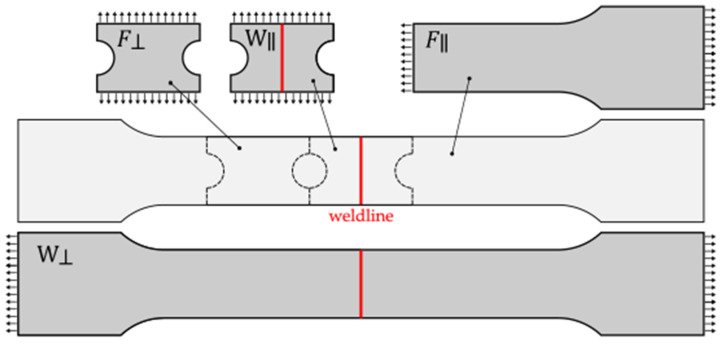
Tensile testing samples and their usage: Whole dumbbell specimen ($W_{⊥}$)—material response perpendicular to weld direction, half dumbbell specimen ($F_{∥}$)—material response in fibre direction, rectangular specimen from the middle ($W_{∥}$)—material response in weld direction, rectangular specimen from the side ($F_{⊥}$)—material response perpendicular to fibre direction.

From these, four different types of specimens were prepared for mechanical characterisation. First, to obtain a stress–strain response perpendicular to the weldline, whole dumbbell specimens ($W_{⊥}$) were used as shown in Figure 5.

Secondly, dumbbell-shaped specimens were cut in half to obtain the stress–strain response of the material in the fibre direction ($F_{∥}$), and lastly, rectangular shaped pieces with side grooves, with dimensions 30 × 20 × 2.3 mm, were extracted from the specimens to characterise the material’s response perpendicular to the fibre direction ($F_{⊥}$) and in the weld direction ($W_{∥}$), as shown in Figure 5.

**Figure 6:**
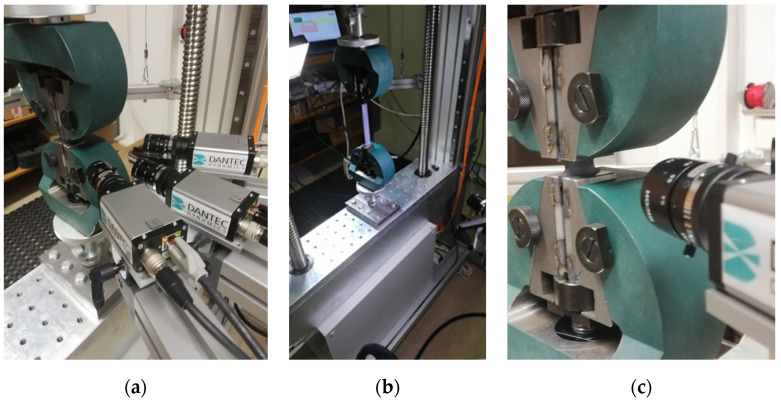
Tensile testing/measuring setup: (**a**) DIC setup; (**b**) whole dumbbell specimen testing; (**c**) rectangular side groove specimen testing.

Tensile tests have been performed at a testing speed of 0.1 mm/s at room temperature (23 °C), on a universal testing machine (in-house developed assembly) where AEP TC4 50 kN load cell and 3 × 5.1 Mpx muti-camera-DIC system Q-400 Dantec Dynamics GmbH, (Ulm, Germany) were utilised for force and strain measurement. The measuring setup with 3 digital cameras is presented in Figure 6.

The DIC evaluation was carried out with Dantec Dynamic Istra 4D (ver. 4.6) software. Technical details of the measuring system and the adopted DIC settings are summarised in Table 2.

**Figure 7:**
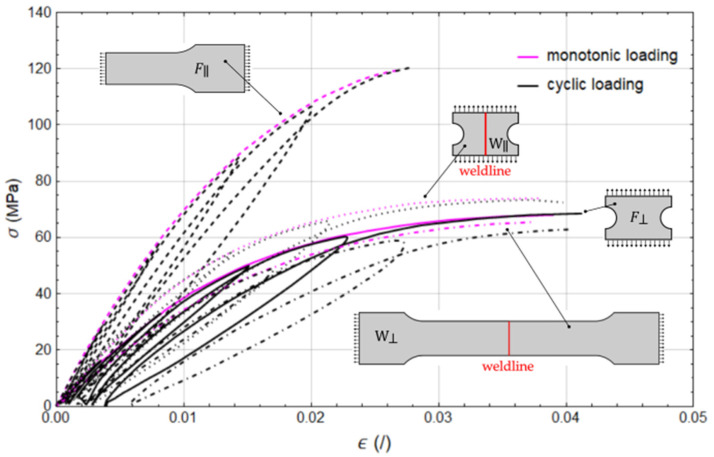
Results of the tensile testing. Whole dumbbell specimen ($W_{⊥}$)—material response perpendicular to weld direction, half dumbbell specimen ($F_{∥}$)—material response parallel to fibre direction, rectangular specimen from the middle ($W_{∥}$)—material response parallel to weld direction, rectangular specimen from the side ($F_{⊥}$)—material response perpendicular to fibre direction.

First, monotonic loading tests were performed for each of the four prepared specimens until failure, followed by a cyclic loading–unloading procedure. The results in terms of stress–strain curves are shown in Figure 7.

### 2.3. Numerical Modelling

One of the critical aspects in the numerical modelling of short fibre-reinforced polymers (SFRPs) is the accurate representation of FOD. Although the highest fidelity can be achieved using X-ray computed microtomography, this technique is typically avoided due to its complexity and often destructive nature. Instead, FOD is commonly estimated through numerical simulation of the injection moulding process.

For the present study, which focuses on numerically evaluating a novel approach to model the material behaviour in weldline regions, we consider injection moulding simulation to be sufficiently accurate. This assumption is supported by two factors. First, the use of simple specimen geometry promotes the formation of a basic weldline, thereby minimising geometric complexity and flow-induced disturbances. This simplification facilitates reliable and straightforward injection moulding simulations. Second, findings from Baradi’s study [16] indicate that even when fibre orientation is precisely captured via microtomography, the simulated stress–strain response in the weldline region still deviates from experimental results. This suggests that additional mechanisms beyond fibre orientation play a dominant role in governing the mechanical behaviour at the weld.

The injection moulding process was simulated using Moldex3D 2025 software. The numerical model of the specimen was discretised using a structured fine finite volume mesh consisting of 168.990 tetrahedral elements. To ensure consistency between simulation and experimental conditions, identical machine settings and processing parameters were applied in the simulation as those used during specimen fabrication (see Table 1).

**Figure 8:**
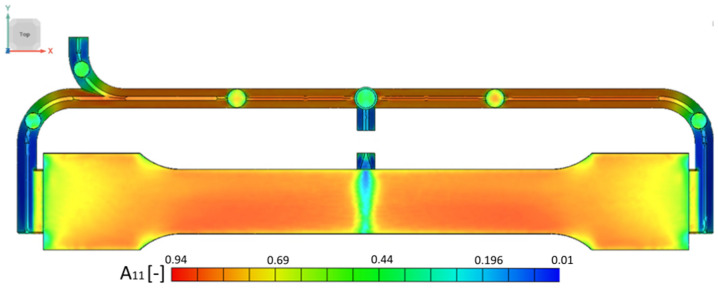
Fibre orientation in x direction.

**Figure 9:**
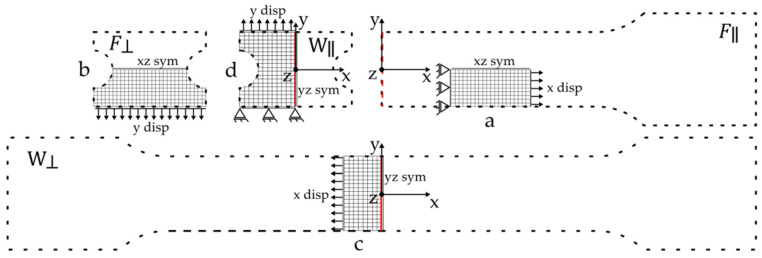
Schematic presentation of the FE models; FE model a—material response parallel to fibre direction ($F_{∥}$), FE model b—material response perpendicular to fibre direction ($F_{⊥}$), FE model c—material response perpendicular to weld direction ($W_{⊥}$), FE model d—material response parallel to weld direction ($W_{∥}$).

The main results of this simulation for our study are the complete fibre orientation tensors *A_ij_* for each integration point of the mesh. Figure 8 presents the fibre orientation results for the most representative fibre alignment in x direction *A*_11_. With the Moldex built-in mapping tool, tensors *A_ij_* were then mapped to the finite element (FE) models prepared for material model calibration and validation. Figure 9 presents geometries and BC for simulating stress–strain behaviour.

These models were prepared with Abaqus 2019 CAE software for each of the specimens used in experimental investigation. Here, we should stress that Abaqus CAE was used for preprocessing only, i.e., to define geometry, mesh, boundary conditions (BCs), and FODs, while the material model and FE solver were prepared in Wolfram Mathematica 12 software. For these models, symmetries and local FODs were taken into account. Models for simulating material response in fibre direction (a) and perpendicular to it (b) avoid the weld and occupy the area where fibres are mostly aligned with the flow direction. Models for simulating material response in perpendicular to weld (c) and in weld (d) direction occupy the area in the middle of the specimen to account for the weld FOD. A structured (regular) 3D mesh with elements of nearly uniform shape and an aspect ratio close to 1 was used. More detailed data on these models can be found in Table 3.

## 3. Results and Discussion

### 3.1. Model Calibration

The presented numerical model has 17 material parameters. For better clarity we summarise them in Table 4.

Not all these parameters are subject to calibration. Approximation of the effective stiffness tensor using the Mori–Tanaka homogenisation scheme requires material properties of the components, which are usually given by the suppliers [27,41]. These are Young’s modulus, Poisson ratios, the fibre aspect ratio and the glass fibre mass percentage (parameters of isotropic base materials in Table 4). It should be noted though that these parameters also allow for some calibration as elastic parameters of sole constituents very likely differ from those inside the composite—for example, material behaviour could be influenced by the degree of crystallisation affected by the presence of fibres in the composite [27], leading to higher stiffness. During preliminary calibration trials, we therefore have a slightly adjusted *E_p_* value (suppliers value 3.3 GPa).

**Figure 10:**
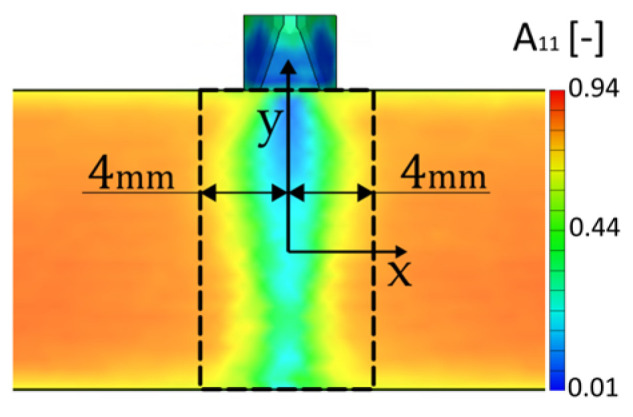
Weldline-influenced area according to the fibre orientation results; in the presented case, $x_{weld}=4\;mm$.

Another parameter determined before calibration is the weldline-influenced area. As presented in Figure 10, we determined $x_{weld}\;$ based on the fibre orientation results as the area with significant change in fibre orientation.

**Figure 11:**
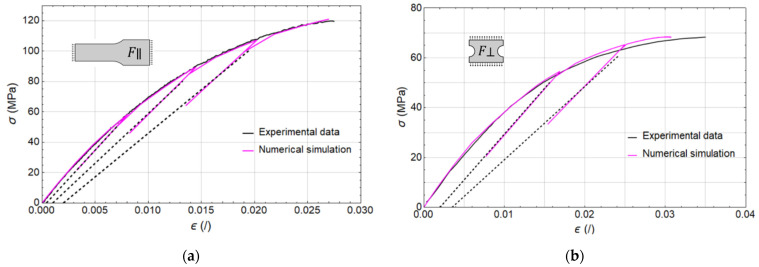
Loading–unloading tensile tests, experimental values (black line), numerical simulations (magenta line): (**a**) Results parallel to fibre direction $F_{∥}$; (**b**) results perpendicular to fibre direction $F_{⊥}$.

Following the initial model setup, a total of ten parameters remained to be calibrated based on the experimental data obtained from the loading tests illustrated in Figure 7. In the first phase, the influence of material damage was disregarded, and six parameters associated with plastic deformation behaviour—designated plasticity in Table 4—were calibrated using monotonic tensile test data from specimens without weldlines ($F_{∥}m$ and $F_{⊥}m$, Figure 7) and corresponding FE models a and b. Subsequently, the two parameters representing damage effects outside the weld region were included and calibrated against the cyclic tensile test data, again from specimens without weldlines ($F_{∥}c$ and $F_{⊥}c$, Figure 7). This necessitated some adjustments to the previously calibrated plasticity parameters to maintain consistency with the observed mechanical response. Final calibrated parameters are enlisted in Table 4, while the corresponding a and b model predictions are presented in Figure 11. Here, the dashed black lines replace the measured hysteresis loops for improved clarity.

**Figure 12:**
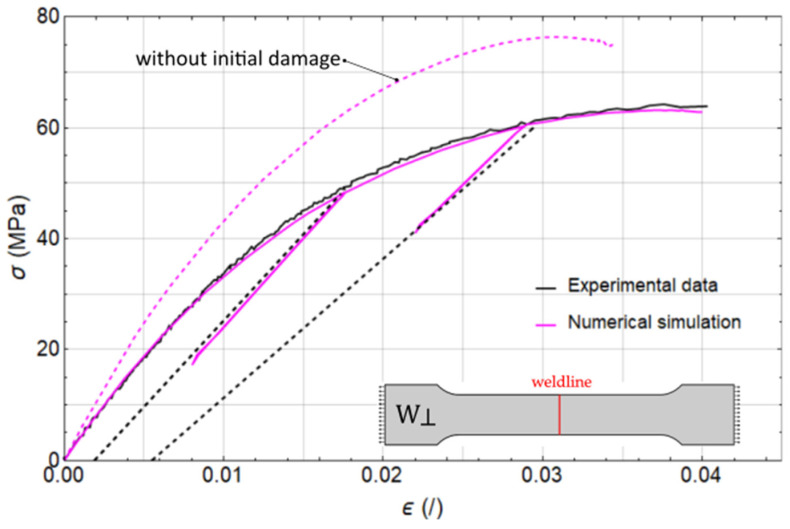
Results of loading–unloading tensile tests perpendicular to weld direction $W_{⊥}$, expermental values (black line), numerical simulations (solid magenta line—with initial damage in the weldline area, dashed magenta line—without initial damage in the weldline area).

In the final stage, the calibration focused on the two parameters governing the initiation and progression of damage within the weld region. These parameters were identified through analysis of the experimental data sets $W_{⊥}m$ and $W_{⊥}c$, Figure 7 and FE model c. The resulting calibration outcomes are presented in Figure 12, with the calibrated parameters listed in Table 4.

Throughout the article, we have consistently emphasised the necessity of constitutively distinguishing the weld region from the surrounding material. Based on the underlying physical phenomena occurring during the injection moulding process, we posit that solely modelling the FOD is insufficient to capture the mechanical behaviour in the weld zone. To address this, we introduced an initial damage parameter specific to the weld region, along with its independent evolution, in order to more accurately represent the localised softening of the material response.

With the completion of the model parameter calibration, we are now able to test our hypothesis. Specifically, by setting the damage parameter in the weld region, $d_{i,weld}$, to zero and assigning the softening parameter $c_{weld}$ a value equal to the failure strain $\varepsilon_{f}$—thereby making the damage in the weld region $d_{\alpha ,weld}$ identical to that in the base material $d_{\alpha }$—the model produces expected overly stiff response, as illustrated in Figure 12 with dashed magenta line. This discrepancy between the measured and simulated material response (without initial damage) perpendicular to the weldline, in fact, underscores the necessity for distinct modelling approaches for the weldline region and the surrounding base material.

### 3.2. Model Validation

To evaluate the validity of the final set of calibrated parameters presented in Table 4, the FE model d was employed to simulate the material response of the weld region under loading parallel to the weldline. Common ground for the specimens and FE models used for calibration is their uniform cross-sections, which carry the applied load. Furthermore, the loading is strictly oriented either parallel or perpendicular to the fibre direction, and the initial damage implemented in FE model c is uniform with respect to the loading axis. These characteristics make them suitable for calibration setup, as they allow for a decoupled identification of the model parameters.

**Figure 13:**
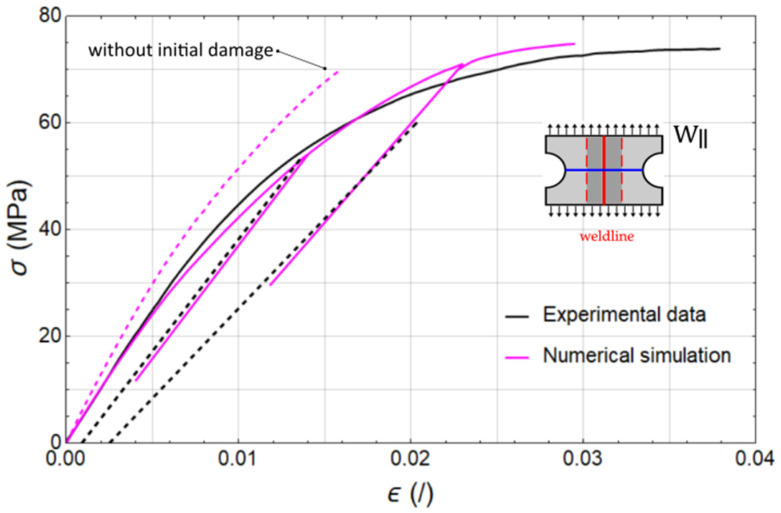
Results of loading–unloading tensile tests parallel to weld direction $W_{∥}$, measured values (black lines), numerical simulations (solid magenta line—with initial damage in the weldline area, dashed magenta line—without initial damage in the weldline area).

In contrast, the specimen ${(W}_{∥}$) and FE model d exhibit a significantly more complex microstructure. In Figure 13, the cross-section responsible for bearing the majority of the load is highlighted with a solid blue line. Tracing this section from the weldline toward either end (due to symmetry), we first encounter fibres aligned with the loading direction, accompanied by gradually decreasing initial damage. Toward the outer boundary of the weldline region—marked with a red dashed line— initial damage decreases to zero, but the fibre orientation transitions to a direction perpendicular to the applied load. This gradual shift in fibre orientation results in a cross-section where each point possesses distinct constitutive properties. Consequently, this specimen provides a suitable basis for validating the calibrated numerical model.

Figure 13 presents a direct comparison between the FE model predictions (magenta line) and the experimentally measured responses (black line).

In Figure 13, we can observe that the elastic response of the material is adequately predicted and from comparison between the measured and simulated slope of the unloading, we can conclude that the same is valid also for damage values at lower strains. There are some deviations present in the middle and upper part of the stress–strain curve but overall, the results of the identification and validation procedures are satisfactory. Certain discrepancies were already present during calibration, such as in the final segment of the stress–strain curve for the material without a weld, loaded transversely to the fibre direction (Figure 11b), where a slightly higher degree of damage would be appropriate. Opportunities for improvement also exist in other cases—namely, the material loaded along the fibre direction (Figure 11a) and the material loaded transversely to the weld (Figure 12). In these instances, the simulated curves show good agreement with the experimental data, although some deviations in damage magnitude remain, as evidenced by differences in the unloading slopes. These discrepancies are most probably also the cause for deviations between simulated and measured results of the validation. However, these could likely be reduced through additional calibration iterations, in which both the damage parameters and plasticity-related parameters are refined.

Since initial damage was not assigned throughout the entire load-bearing cross-section of the validation specimen, an additional simulation was performed to predict the material response in the absence of initial damage (represented by the dashed magenta line in Figure 13). As anticipated, the results reaffirm the hypothesis outlined in the introduction: within the weld region, it is necessary not only to account for fibre orientation but also to apply constitutive corrections which can be applied through incorporation of initial damage. This approach enables the modelling of reduced load-bearing capacity caused by various phenomena occurring during injection moulding at the weld interface—such as poor molecular bonding, void formation, surface notches, and molecular alignment perpendicular to the flow direction.

The proposed methodology was guided by the principle of simplicity to facilitate practical application to real-world cases. As currently formulated, it is feasible to extract the specimens required for parameter identification directly from actual components, based on fibre orientation data obtained from injection moulding simulations. This is where DIC plays a central role—it enables full-field strain measurements across various types of test specimens. In our case, this proved particularly beneficial, as it allowed for direct comparison of deformations within the region covered by the FE models, eliminating the need to simulate the entire specimen geometry.

To streamline implementation, the weld was positioned at the origin of the coordinate system, thereby confining initial damage to the weldline. While this assumption is effective for single-weld scenarios, it becomes impractical in components with multiple welds. This limitation can be elegantly addressed by introducing a field variable derived from injection moulding simulation data, ranging from zero to one—where one corresponds to the weld centre and zero to its periphery. Alternatively, a reduction factor for elastic properties at the weld location, already obtainable from injection moulding simulation software, can be used. By reformulating Equation (21) to depend on this field variable rather than spatial coordinates, initial damage can be assigned across multiple weld regions. Furthermore, such field variables can be dependent also on other parameters that can influence initial damage, such as the pressure in the melt, allowing for weld-specific damage characterisation. Additionally, such an implementation could also mean further improvement in the predictive capabilities of the model. In its current formulation a uniform initial damage is imposed across cross-sections parallel to the weldline. By introducing a field variable dependence for the initial damage, we might achieve more realistic spatial distribution and improved stress–strain results.

Another potential enhancement of the presented model involves incorporating its sensitivity to the injection moulding process window. Currently, model parameters are calibrated solely against stress–strain data from specimens produced under the conditions listed in Table 1. However, variations in processing parameters affect weldline integrity. Elevated temperatures—above the crystallisation point for semi-crystalline polymers and the glass transition for amorphous ones—promote interlinking and macromolecular diffusion at the weld area [6]. Increased injection pressure minimises void formation and surface distortions [9]. Moreover, the temporal profiles of temperature, pressure, and other field variables in the weld zone are governed by parameters such as melt and mould temperature, packing pressure, and injection speed [9,11,15,42,43,44,45,46]. Future work should therefore aim to establish quantitative relationships between processing conditions and weldline damage parameters to improve the model’s applicability.

## 4. Conclusions

In this study, a hybrid micro–macromechanical constitutive model was extended to account for localised initial damage in weldline regions of SFRPs. The model integrates fibre orientation data from injection moulding simulations with a phenomenological damage formulation, enabling spatially resolved predictions of mechanical behaviour. Calibration and validation were performed using directional tensile tests on polyamide 66 specimens reinforced with 25 wt% glass fibres, with digital image correlation (DIC) employed to capture full-field strain distributions.

The results demonstrate that incorporating initial damage and its independent evolution within the weld zone significantly improves the accuracy of mechanical response predictions. Simulations aligned well with experimental data, particularly in capturing the softening behaviour and reduced load-bearing capacity in weld-affected regions. The model also maintained computational efficiency and compatibility with standard finite element workflows. Minor discrepancies were observed in unloading slopes and damage magnitude, suggesting potential for further refinement, but overall, the approach proved robust and reliable for structural assessment of SFRP components.

Future work should focus on generalising the model for components with multiple welds by introducing a field variable that captures weld location and severity. This variable depends mostly on fibre orientation but can also be influenced by process parameters such as melt pressure or molecular diffusion indicators. Additionally, expanding the model to incorporate temperature-dependent behaviour and fatigue damage would enhance its applicability in long-term performance simulations and safety-critical design scenarios.

## Figures and Tables

**Table 1:** Injection moulding parameters.

Melt Temperature [°C]	Mould Temperature [°C]	Injection Pressure [bar]	Screw Speed [mm/s]	Packing Pressure [bar]
290	54	550	30	410

**Table 2:** Details of the DIC measuring system.

Company	Dantec Dynamics GmbH (Ulm, Germany)
Model	Q-400
Cameras	Manta G-507 (3 pieces)
Image resolution	2464 × 2056 pixel
Objective focal distance	35 mm
Field of view	approx. 100 mm × 25 mm ^(1)^, 70 mm × 25 mm ^(2)^, 15 mm × 25 mm ^(3)^
Patterning technique	matt white spray paint speckles
Pattern feature size	approx. 3 pixels
DIC software	DANTEC Dynamics, Istra 4D (ver. 4.6)
Facet size	19 pixels
Grid spacing	12 pixels
Spatial smoothing	none
Temporal smoothing	none
Number of acquired data points	7500 ^(1)^, 3300 ^(2)^, 4000 ^(3)^

^(1)^ Whole dumbbell specimen ($W_{⊥}$), ^(2)^ half dumbbell specimen ($F_{∥}$), ^(3)^ rectangular specimens ($W_{∥}$) and ($F_{⊥}$).

**Table 3:** FE models data.

FE Model	Sample (Figure 5)	Dimension [mm]	Discretisation C3D8R	Mesh Size [mm]	Symmetry Plane
a	$F_{∥}$	20 × 10 × 2.3	975	0.8 (3 layers across section)	xz
b	$F_{⊥}$	30 × 10 × 2.3	1104	0.8 (3 layers across section)	xz
c	$W_{⊥}$	4 × 20 × 2.3	1600	0.5 (5 layers across section)	yz
d	$W_{∥}$	15 × 20 × 2.3	5370	0.5 (5 layers across section)	yz

Again, we should point out that models a, b, and c were used for model calibration, while model d was used for verification.

**Table 4:** All material parameters of the model.

Parameter	Value	Parameters of/for
*E_p_*: Polyamide 66 Young’s modulus [GPa]	3.7 ^(1)^	isotropic elasticity of base materials
*ν_p_*: Polyamide 66 Poisson’s ratio	0.3 ^(1)^	
*E_f_*: Glass fibre Young’s modulus [GPa]	70 ^(1)^	
*ν_f_*: Glass fibre Poisson’s ratio	0.2 ^(1)^	
*λ*: Glass fibre aspect ratio	28 ^(1)^	
*m_f_*: Glass fibre mass percentage [%]	25 ^(1)^	
$\sigma_{y}$: hardening parameter [MPa]	50 ^(2)^	plasticity
*h*: hardening parameter [MPa]	1400 ^(2)^	
*q*: hardening parameter	0.45 ^(2)^	
*F*: Hill’s yield criterion parameter	2.4 ^(2)^	
*G*: Hill’s yield criterion parameter	0.42 ^(2)^	
*M*: Hill’s yield criterion parameter	1.5 ^(2)^	
$\varepsilon_{f}$: failure strain	0.15 ^(3)^	damage outside the weld area
*g*: damage evolution exponent	0.58 ^(3)^	
$x_{weld}$: weldline-influenced area [mm]	4 ^(4)^	damage in the weld area
$d_{i,weld}$: initial weld damage	0.43 ^(5)^	
$c_{weld}$: weld damage evolution	0.833 ^(5)^	

^(1)^ Provided by the suppliers of the material, ^(2)^ plasticity parameters calibrated in the first step to monotonic tensile tests of the base material, ^(3)^ damage parameters calibrated in the second step to cyclic tensile tests of the base material, ^(4)^ estimated from injection moulding simulations, ^(5)^ initial damage parameters calibrated in the third step to the cyclic tensile test perpendicular to the weld.

## Data Availability

The original contributions presented in the study are included in the article, further inquiries can be directed to the corresponding author.

## Abbreviations

The following abbreviations are used in this manuscript:

SFRP	Short fibre-reinforced polymer
FOD	Fibre orientation distribution
FE	Finite element
DIC	Digital image correlation
